# Midwifery in 1919-1920

**Published:** 1921-01-22

**Authors:** 


### Midwifery in 1919-1920.

An unusually interesting report has been issued relating to the work of the Central Midwives Board for the year ended March 1920, but we cannot forbear the criticism that it is a rather tardy arrival, over nine months after the conclusion of the period covered. The statistics are prepared with the usual exact care, and show but slight variations on previous years. The Midwives' Roll contains the names of 45,960 women, but the impossibility of getting, in all cases, notification of death detracts from the value of these figures. The Board is on surer ground in its computation of the number of practising midwives, who now total 11,488, of whom 68.5 per cent. are trained; the untrained are now reduced to 3,623, or 31.5 per cent. The number of training-centres at institutions and homes in England and Wales stands at 159, of which sixty-three are Poor-law institutions. A valuable appendix to the report gives the names of the institutions and the number each of them has trained, with the number of these midwives practising in 1919. From this it appears that a very small proportion of Poor-law nurses take up the practice of midwifery. Taking the figures for Great Britain and Ireland only 179 practising midwives represent the 2,763 midwives trained under the Poor Law, a percentage of 6.5 per cent. Of the midwives enrolled by approved institutions (other than Poor Law) the percentage practising is 25.3, the number reaching a total of 5,603 out of 22,181. This is a very remarkable contrast, of which we fail to apprehend the cause. An encouraging feature in this survey of the midwifery field is the inclusion of four approved institutions in India (two in Bombay, one in Calcutta, and one in Madras) and one in China (Hong Kong), the number trained being thirty-seven, of whom, alas! but three are practising. Of midwives privately trained the percentage practising is 25.6.

The percentage of failures, taken all together, fell from 19.9 to 18.8. It is very unequally distributed among the centres, where it varied from 11.3 at Newcastle-on-Tyne to 29.4 at Liverpool and 28.9 at Manchester. The London percentage was 17.8. This year the number of examinations has been raised, in response to representations made to the Board by the heads of training institutions. They will take place in alternate months, starting with February, and will be held at London, Liverpool, Manchester, Leeds, Newcastle, Birmingham, and Bristol, in such an order that each of the provincial centres will have three examinations, and London six, in the year. New rules for the better promotion of breast feeding, by providing for the notification of each case in which it is proposed to substitute artificial feeding, have been approved by the Privy Council. The Board has very wisely refused to limit the number of cases taken by a midwife in the course of the year.

